# Supplementary material for: Isolation and Characterization of the Lytic Pseudoxanthomonas kaohsiungensi Phage PW916
Source: Viruses. 2022 Aug 2;14(8):1709. doi: 10.3390/v14081709 (PMC9414467; doi:10.3390/v14081709)
Supplement: Supplementary file 1 [file viruses-14-01709-s001.zip › viruses-1812392 - supplementary.pdf]

viruses

MDPI

Supplementary Data

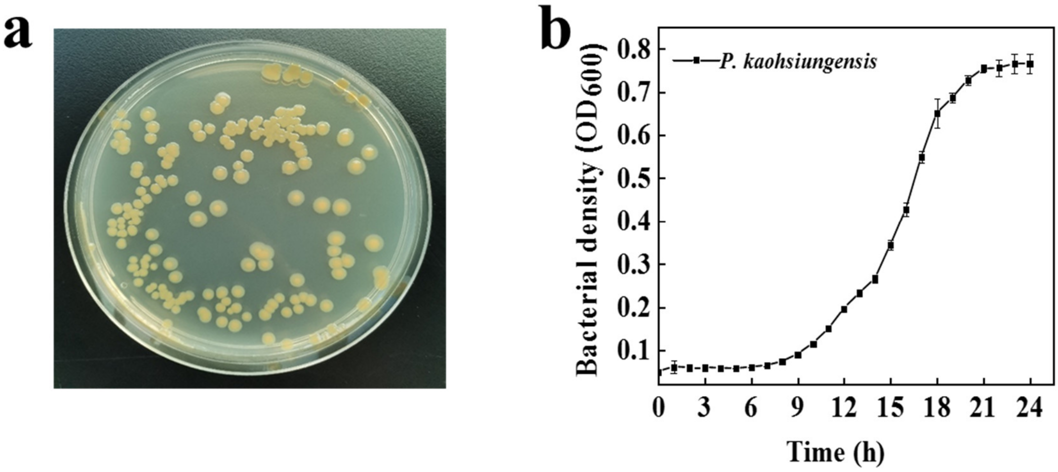

Figure S1. Biological features of host. (**a**) Colonial morphology of *P. kaohsiungensis*. (**b**) Growth curve of *P. kaohsiungensis*.

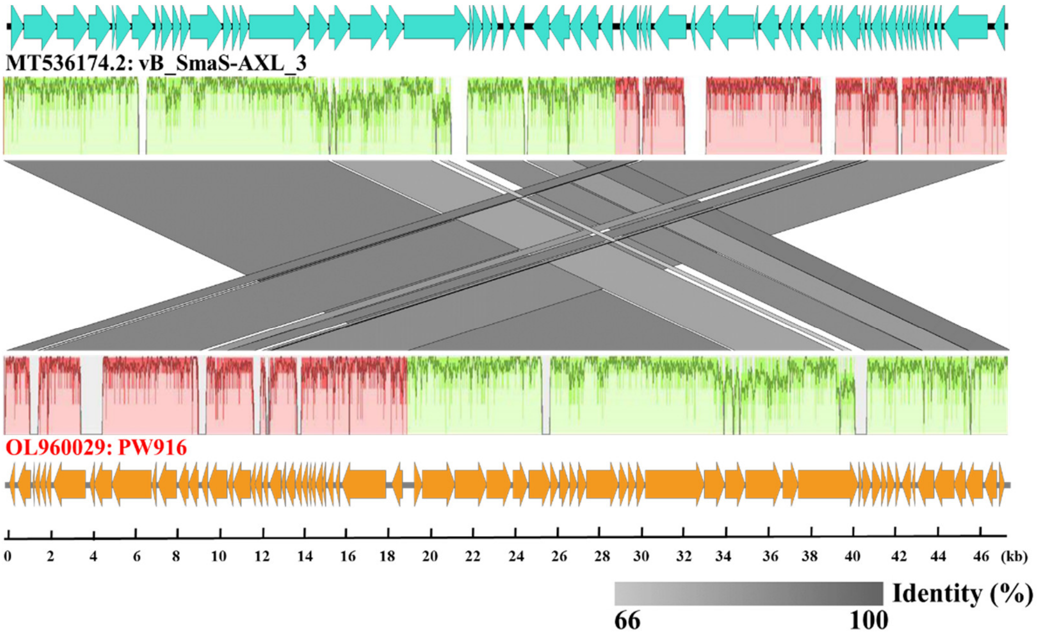

Figure S2. Genomic comparison between phage PW916 and Caudoviricetes phage vB_SmaS-AXL_3 (RefSeq accession number MT536174.2). Arrows represent open reading frames (ORFs) of the phage and gray bands denote the similarity in nucleotide sequences between genomic regions.

Table S1. Minimum inhibitory concentration (MIC) of *P. kaohsiungensis.*

Antibiotic Name	MICs (μg/mL)	Antimicrobial Susceptibility Tests of CLSI (μg/mL))
Tetracycline	0.42	4
Chloramphenicol	1.64	16
Ampicillin	3.41	100
Kanamycin	121.3	100
Amoxicillin	74.6	64
Streptomycin	46.55	30
Rifampin	1.87	100

Table S2. Comparative analysis of phage PW916 with IMG/VR database based on genome.

Genome ID	Subject Length(bp)	Estimated Completeness (%)	Identities (%)	Phage Lineage	Predicted Host Lineage
3300014059	41149	100	84	Caudovirales;	Hydrogenophilales
3300025115	38459	100	79	Caudovirales;	-
3300001594	129024	100	73	Caudoviricetes;	Thioalkalivibrio
3300008339	37900	100	77	Caudovirales	-
3300003972	45492	100	88	Caudoviricetes	-
3300000227	34746	100	78	Caudovirales	-
3300008266	37900	100	77	Caudovirales	-
3300001592	129042	100	73	Caudoviricetes	Thioalkalivibrio;
3300008450	37900	100	77	Caudovirales	-
3300020156	47991	100	76	Caudoviricetes	-
3300008451	37900	99.66	77	Caudovirales	-
3300020711	38318	98.03	78	Caudovirales	-
3300025779	38287	97.96	78	Caudovirales	-
3300027111	50204	97.92	88	Caudoviricetes	Sinorhizobium;
3300002220	40879	95.38	73	Caudovirales	Thioalkalivibrio
3300005898	38045	83.20	71	-	-
